# Supplementary material for: The causal role of circulating inflammatory markers in osteoporosis: a bidirectional Mendelian randomized study
Source: Front Immunol. 2024 Jul 18;15:1412298. doi: 10.3389/fimmu.2024.1412298 (PMC11291241; doi:10.3389/fimmu.2024.1412298)
Supplement: Supplementary file 1 [file DataSheet_1.zip › supplementary material/CIM-OSTEOPOROSIS /GCST90274811.csv_leave_one_out.pdf]

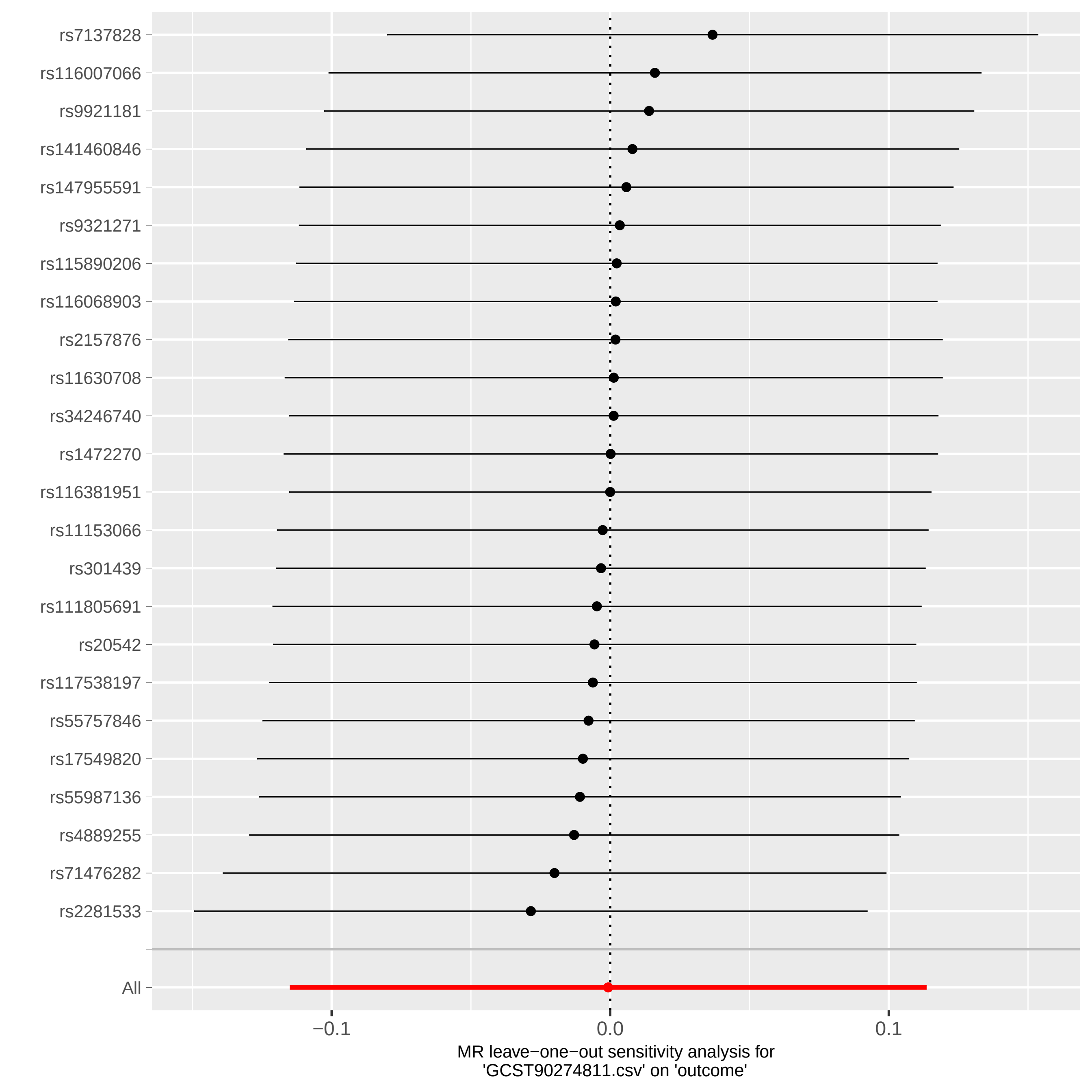

rs7137828
rs116007066
rs9921181
rs141460846
rs147955591
rs9321271
rs115890206
rs116068903
rs2157876
rs11630708
rs34246740
rs1472270
rs116381951
rs11153066
rs301439
rs111805691
rs20542
rs117538197
rs55757846
rs17549820
rs55987136
rs4889255
rs71476282
rs2281533
All
−0.1
0.0
0.1
MR leave−one−out sensitivity analysis for
'GCST90274811.csv' on 'outcome'
